# Mathematical modelling of *WOX5*- and *CLE40*-mediated columella stem cell homeostasis in *Arabidopsis*

**DOI:** 10.1093/jxb/erv257

**Published:** 2015-05-26

**Authors:** Sarah Richards, Rene H. Wink, Rüdiger Simon

**Affiliations:** ^1^Institute of Developmental Genetics, Heinrich Heine University, 40225 Düsseldorf, Germany

**Keywords:** Columella cells, CLE40, gene regulatory networks, peptide signalling, root development, stem cell homeostasis, WOX5.

## Abstract

The root meristem of *Arabidopsis thaliana* harbours a pool of stem cells, which divide to give rise to the differentiated cells of the various root tissues. Regulatory networks of inter-cellular signalling molecules control the homeostasis of stem cell number and position so that both stem and differentiated cells are consistently available. This work focuses on the transcription factor *WUSCHEL-RELATED HOMEOBOX 5* (*WOX5*), the signalling peptide *CLAVATA3/EMBRYO-SURROUNDING REGION 40* (*CLE40*) and the feedback loops involving them, which maintain the columella stem cells (CSCs). WOX5 signals from the quiescent centre (QC) to promote stem cell fate, while CLE40 is secreted from the differentiated columella cells (CCs) to promote differentiation. Our analyses of mathematical models of this network show that, when cell fate is controlled primarily by antagonistic factors, homeostasis requires a spatial component and inter-cellular signalling. We have also found that WOX5 contributes to, but is not absolutely necessary for, CSC maintenance. Furthermore, our modelling led us to postulate an additional signalling molecule that promotes CSC maintenance. We propose that this WOX5-independent signal originates in the QC, is targeted by CLE40 signalling and is capable of maintaining CSCs.

## Highlight

An unknown columella stem cell (CSC)-regulating factor subject to regulation by CLE40 can maintain CSCs in the absence of WOX5. Mathematical modelling of CSC homeostasis highlights importance of intercellular signalling.

## Introduction

The root stem cell niche of *Arabidopsis thaliana* is a collection of undifferentiated cells which divide to give rise to the many different root cell types. In the centre of the niche is the quiescent centre (QC), a group of four cells which maintain the identity of the stem cells and utilize marginal cell division activity to replenish the stem cell supply. The stem cells proximal to the QC are the vascular initials, and the stem cells lateral to the QC are initials for the endodermis, epidermis and lateral root cap. Those distal to the QC are the columella cell initials, also called columella stem cells (CSCs). Their descendants, the columella cells (CCs), are located distal to the CSCs and they detect the direction of gravity, store energy by accumulating starch and provide a protective layer for the stem cell niche.

The stem cell niche is made up of the QC cells and one layer of adjacent stem cells surrounding it. The cells in this layer serve as initials for all of the cell types in the various root tissues; when a stem cell divides, the cell in contact with the QC remains a stem cell while the other enters a differentiation pathway. The differentiated cell then fulfills particular tasks necessary for the plant’s development and function. Stem cell homeostasis (a steady number of stem cells) is necessary so that there is a supply of both the differentiated cells and their stem cell initials (Stahl and Simon, 2005).

**Fig. 1.**
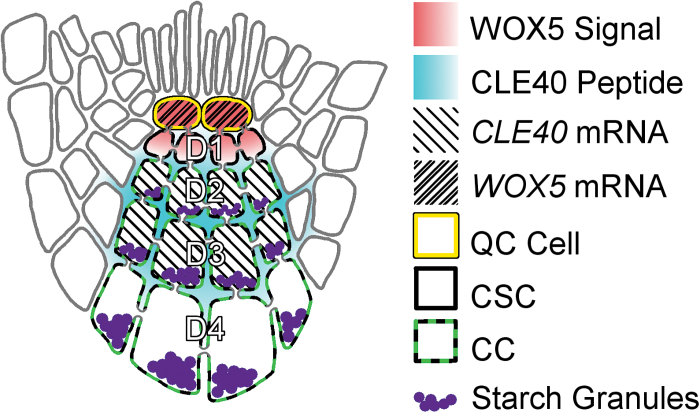
WOX5 signal and CLE40 peptide locations and their effect on CSC fate. *WOX5* is expressed in the QC and signals to maintain CSCs distal to the QC. *CLE40* is expressed in CCs, the differentiating daughters of the CSCs, and secreted into the apoplast, where it can act through plasma membrane-localized receptor-like kinases and inhibit the WOX5 signal. CCs are identifiable by their stainable starch granules.

If the QC is ablated, the stem cells around it differentiate. The QC then reforms in a location proximal to the original QC site and a new stem cell niche is formed. This suggests that the surrounding stem cells are maintained by short-range signalling from the QC (van den Berg *et al.*, 1997). The number and position of the CSCs are regulated by signals from both the QC and CCs. The QC expresses the transcription factor *WUSCHEL-RELATED HOMEOBOX 5* (*WOX5*), which promotes CSC fate, while the CCs express and secrete the signalling peptide CLAVATA3/EMBRYO-SURROUNDING REGION 40 (CLE40) to encourage differentiation into CCs (Sarkar *et al.*, 2007; Stahl *et al.*, 2009). A typical wild-type *Arabidopsis* root has one layer of CSCs in the first layer distal to the QC (D1). The cells from D2 to the root tip have starch granules, the trait used to distinguish between CC and CSC identity (Fig. 1). It has been shown that constitutive expression of *WOX5* results in massive accumulation of CSCs, while *wox5-1* loss-of-function mutants typically have starch in the D1 layer. This suggested that WOX5 is both necessary and sufficient for CSC maintenance (Sarkar *et al.*, 2007).

WOX5 is a homologue of the transcription factor WUSCHEL (WUS) which is expressed in the organizing centre of the shoot apical meristem (Sharma *et al.*, 2003; Sarkar *et al.*, 2007). WUS is a mobile protein which regulates cell fate non-cell-autonomously and expression of WUS in place of WOX5 can functionally replace WOX5, suggesting that both proteins perform very similar functions at opposite ends of the plant (Yadav *et al.*, 2011). However, how *WOX5* acts from the QC to regulate CSC fate in the neighbouring distal cells and whether WOX5 is also a mobile protein is not yet known. It is assumed that either *WOX5* itself, or a gene transcriptionally controlled by WOX5, gives rise to a signal that moves from the QC to the CSCs to maintain their cell fate. This WOX5-dependent signal (W) could be the WOX5 protein moving from the QC through plasmodesmata to promote stem cell fate in adjacent cells (as WUS does in the shoot), or it could be a mobile signal generated by WOX5 that diffuses to the next cell layer (Sarkar *et al.*, 2007).

Acting antagonistically to the *WOX5*-mediated promotion of CSCs, *CLE40* is expressed in the CCs themselves to promote differentiation. CLE40 is secreted into the inter-cellular space and interacts with receptor-like kinases (RLKs) ARABIDOPSIS CRINKLY4 (ACR4) and CLAVATA1 (CLV1), embedded in the plasma membrane of neighbouring cells, to limit *WOX5* expression. Intriguingly, both ACR4 and CLV1 preferentially localize to plasmodesmata, where they may directly regulate the mobility of QC-derived stemness factors in a CLE40-dependent manner (Stahl *et al.*, 2013). In *cle40-2* mutants the *WOX5* expression domain is expanded, while the differentiation of CSC descendants is delayed, often resulting in the maintenance of two stem cell layers. The addition of synthetic CLE40 peptide (CLE40p) decreased the number of stem cells in a dose-dependent manner. Addition of CLE40p to wild-type roots promoted differentiation to CCs. There is a substantial difference in the phenotypes of wild type and *wox5-1* mutants when sufficient CLE40p is added; with the same dosage of CLE40p, the *wox5-1* mutants have a higher frequency of starch granules in the QC position. These results together indicate that CLE40 promotes differentiation via two pathways, one independent of WOX5 and the other via WOX5 (Stahl *et al.*, 2009).

WOX5 and CLE40 are part of a subnetwork within the larger network of QC and CSC fate-governing factors. *WOX5* expression is downstream of several pathways involving the auxin maximum around the QC (Ding and Friml, 2010). *WOX5* expression also relies on a transcription factor *SCARECROW (SCR*), which, similarly to *WOX5*, is expressed in the QC, specifies QC identity and maintains stem cells. Both WOX5 and SCR can function redundantly to maintain the cortex initials proximal to the QC (Sarkar *et al.*, 2007). Several other transcription factors have been reported to control CSC abundance, including auxin response factors (ARF) ARF10 and ARF16 (Wang *et al.*, 2005) and a regulatory feedback loop between the NAC-domain proteins FEZ and SOMBRERO (SMB; Willemsen *et al.*, 2008). Whereas ARF10 and ARF16 were suggested to restrict CSC fate in a parallel pathway to WOX5, it was hypothesized that SMB could negatively regulate WOX5 via the CLE40/ACR4 receptor module (Bennett *et al.*, 2014). The auxin responsive protein IAA17 was also shown to indirectly regulate CSC fate through mediation of the auxin response in the QC, which is crucial for WOX5 activity (Tian *et al.*, 2014).

Here we used mathematical modelling to test whether the current information available on WOX5 and CLE40 and their known interactions can explain in sufficient detail the observed cell fates in different mutant backgrounds and upon experimental changes of the amount of individual components. We have developed three mathematical models for the CSC fate-governing regulatory network consisting of WOX5 and CLE40. We determined that a single-cell model of this network, which lacked signals from other cells, was incapable of describing the observation of long-lived CSCs. Although a first multi-cell model of the network was sufficient to simulate the majority of biological tests, only a modified multi-cell model introducing an additional stem cell promoting factor into our network was able to describe all experimental results.

## Materials and methods

### Model and simulations

All model equations were solved using Matlab function ode45, which provides solutions for ODEs at discrete time points. Parameter values were scaled by hand.

For the multi-cell models, solutions were obtained for time *t* from 0 to 100, where values of the variables were compared at *t*=99 and *t*=100. If the values differed by more than 1e-5, the simulations were run for a longer time. The values of the variables at the last time point were used to determine cell fate.

### Plant accessions

*Arabidopsis thaliana* ecotype *Columbia* (Col-0) was used as wild type. Col-0 was the background for all mutant seeds. *wox5-1* mutant seeds (SALK_038262) were obtained from the Nottingham *Arabidopsis* Stock Centre (NASC, UK) and were described in Sarkar *et al.* (2007). *cle40-2* mutants were previously described (Stahl *et al.*, 2009). Homozygous *cle40-2/wox5-1* double mutants were generated via crossing and verified by genotyping.

### Plant growth conditions

Plant growth conditions were previously described in Stahl *et al.* (2009).

### Starch staining and microscopy

Starch granules were stained with the mPS-PI method described in Truernit *et al.* (2008) and imaged with a Zeiss LSM 510 confocal microscope.

## Results and discussion

### Spatial protein distribution is important for CSC patterning

The analysis of a network with several components and relationships requires at least some known parameter values. While a mathematical model can be used to predict the values of variables (e.g. concentration of CLE40), parameters are set ahead of time and remain constant throughout simulations. Examples of parameter values include production and degradation rates of CLE40. A model may exhibit very different behaviours based on small changes in the value of a parameter. It is possible to fit a model of a network to data and determine likely values of some of the parameters from the fitting (Gutenkunst *et al.*, 2007). To model CSC regulation, it would be useful to find the concentrations of WOX5, CLE40 and the other factors affecting CSC fate, but measuring the concentrations of WOX5 and CLE40 *in vivo* has proven to be a technical challenge. Consequently in this case, it would be best to use the simplest model with the fewest possible regulatory factors to elucidate the roles of WOX5 and CLE40 in cell fate determination. This way, the number of parameters is manageable, and it is possible to analyse the model output for many different sets of parameter values. This leads to knowledge about the model’s general behaviour and, consequently, a possibility of insight into the function of the network emulated by the model.

**Fig. 2.**
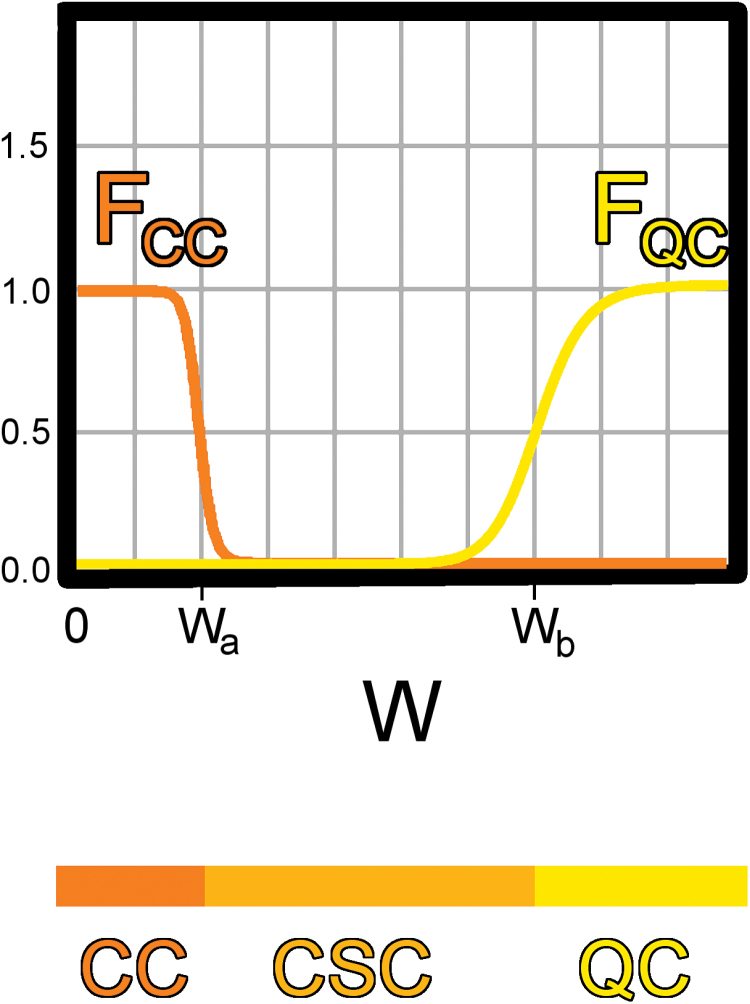
Values of W determine cell fate, represented by F_CC_ and F_QC_. The numerical representatives of CC and QC fate (F_CC_ and F_QC_ respectively), were modelled as Hill functions dependent on W. Parameters W_a_ and W_b_ determine their half-maximum values. Cells with values of W<W_a_, W_a_<W<W_b_, and W>W_b_ would be classified as CC, CSC and QC cell, respectively.

To that end, we first developed a simple single-cell model, where the fate of a cell is controlled entirely by the interactions and different concentrations of a *WOX5*-derived signal (W) and the signalling peptide CLE40 (C). To keep the model simple, we assumed that everything necessary for the function of W and C (e.g. RLKs necessary for signal transmission) was abundant. In order to incorporate QC, CSC and CC fates into a mathematical model, numerical representatives of cell fate were required. These representatives could then be used to specify production rates of the two factors W and C, allowing us to require C to be produced by CCs, but not by CSCs or the QC. The representatives could also determine the fate of a cell, so that cells with insufficient concentrations of W differentiate into CCs as they do under experimental conditions. We designated variables F_CC_ and F_QC_ as the representatives, with high values of F_CC_ representing CC fate and high values of F_QC_ representing QC fate. Both F_CC_ and F_QC_ vary between 0 and 1 (Fig. 2) as a function of W. Parameters W_a_ and W_b_ are the half-maximum values of F_CC_ and F_QC_, respectively. The value of F_QC_ increases with W concentration, simulating the WOX5-dependent aspects of QC fate. A cell with W>W_b_ would be categorized as a QC cell. Low values of W yield higher values of F_CC_, simulating differentiation to CC fate when W concentration is insufficient for CSC maintenance. We would categorize a cell with W<W_a_ as having CC identity and a cell with any value of W between W_a_ and W_b_ as having CSC identity.

**Fig. 3.**
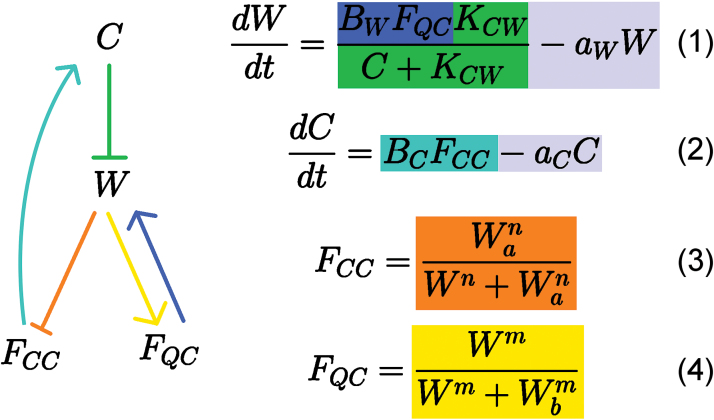
Graphical and mathematical presentation of the relationships between C, W, F_CC_ and F_QC_. Equations (1) and (2) determine the rate changes of W and C values, respectively, while equations (3) and (4) detail the Hill functions governing cell fate dependence on W. C inhibits W (green), while W promotes production of F_QC_ (yellow) and represses F_CC_ (orange). F_QC_ provides positive feedback to W (blue), and F_CC_ promotes C (teal). W and C are degraded at a constant rate (grey). B_w_, K_cw_, a_w_, B_c_, a_c_, W_a_, W_b_, m and n are parameters.

**Fig. 4.**
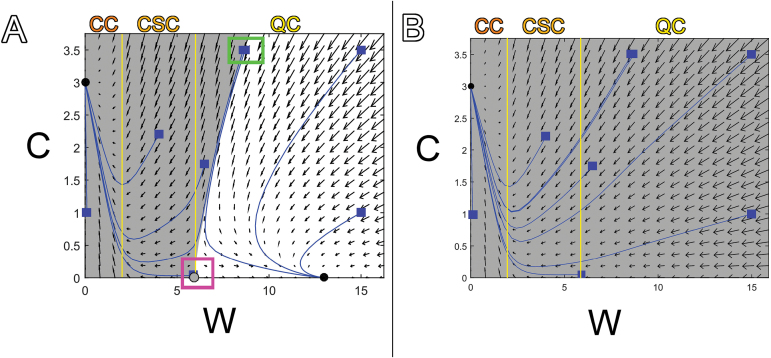
Vector diagram of single-cell model results. (A) Fixed points are represented by black and grey filled circles, stable and unstable respectively. Solutions (blue lines) over time were obtained from various initial values (blue squares). Initial values chosen close to the unstable fixed point (like the initial value in the pink box) yield solutions that flow away from that fixed point. The basin of attraction for the CC fixed point at coordinates (0, 3) is shown in grey, and the basin of attraction for the QC fixed point close to (13, 0) is shown in white. Two initial values close to one another but lying in separate basins of attraction (green box) lead to different solutions over time. Vertical yellow lines mark changes in cell fate (where W=W_a_ and W=W_b_). (B) A different set of parameter values (identical to the values used to produce Fig. 1A, but with decreased maximum production rate of W) yields only one fixed point (black dot), which is stable. All values of W and C are within its basin of attraction (grey).

F_QC_ as the representative of QC fate determines the production rate of W. To simulate production of W only in QC cells, the W production rate increases as F_QC_ increases (blue in Figs 3 and 4). Likewise, F_CC_ determines the production rate of C. The production rate of C increases as F_CC_ increases to simulate CLE40 being produced only in CCs (teal in Figs 3 and 4). A network diagram of the interactions is shown in Fig. 3 with the corresponding equations.

A table of parameter symbols and definitions can be found in Table 1. The parameter values have units, but the individual parameter values are not reliable for comparison with measurements. Determining appropriate parameter values would require numerical data, and the experimental results used to calibrate the model are qualitative in nature.

**Table 1.** Parameter, variable, and index symbols and definitions This is a comprehensive list for all models presented in this paper, so specification of which model uses each parameter, variable and index is provided in the Models column.

**Symbol**	**Definition**	**Models**
W, W_i_	Concentration of WOX5-derived signal, concentration of W in cell i	All
C, C_i_	Concentration of CLE40 peptide, concentration of C in cell i	All
X_i_	Concentration of X in cell i	W/C/X
F_CC_, F_CC,i_	Numerical representative of CC fate, F_CC_ in cell i	All
F_QC_	Numerical representative of QC fate	Single-cell
S_i_	Sum of W_i_ and X_i_	W/C/X
B_W_, B_C_, B_X_	Maximum production rates of W, C, and X	All
a_W_, a_C_, a_X_	Degradation rates of W, C, and X	All
K_CW_	Value of C where W production rate is halved	All
K_CX_	Value of C where X production rate is halved	W/C/X
W_a_	Value of W between CC and CSC fate	All
W_b_	Value of W between CSC and QC fate	Single-cell
S_a_	Value of S_i_ needed to maintain CSC fate	W/C/X
n	Hill co-efficient which controls steepness of S_1_ curve	All
m	Hill co-efficient which controls steepness of S_2_ curve	Single-cell
D_w,i_, D_c,i_, D_x,i_	Proportional to diffusion rate of W, C, X between cell i-1 and cell i	Multi-cells
D_w,i+1_, D_c,i+1_, D_x,i+1_	Proportional to rate of diffusion of W, C, X between cell i and cell i+1	Multi-cells
i	Cell index	Multi-cells

To explore what would happen to cells with various initial values of W and C, the model given by the equations in Fig. 4 was implemented in a Matlab program (Supplementary File S1). It includes a graphical user interface so that a user can easily edit the parameter values, give initial values for W and C in the cell and see the model output. We compiled several results from different sets of initial values (Fig. 4A). The same was done for a different set of parameter values (Fig. 4B), which illustrates how the model predictions can change when parameter values are altered. The solutions are shown on top of a vector diagram. The vectors in the vector diagram point in the direction of change of W and C. Values of W and C start at the given initial value (blue squares in Fig. 4) and change over time in the direction of the arrows until they reach a stable fixed point (black dots in Fig. 4). The stable fixed point comprises the coordinates of W and C values at a stable equilibrium solution (i.e. cell fate). The results of this model yield at most two stable fixed points (or fates) for the cell, one with no W and a fixed amount of C (CC fate), and another with a small amount of C and a large amount of W (QC fate). When these two stable fixed points are present, there is always an unstable fixed point as well within the CSC domain (grey dot in Fig. 4A). However, we do not count the unstable fixed point as CSC fate. In order for a fixed point to represent a cell fate, values of W and C must be able to settle on that fixed point. Unlike the stable fixed points, which have arrows pointing toward them, unstable fixed points only have arrows pointing away; solutions that start close to a fixed point always flow away from it over time, never toward it (green box in Fig. 4). Therefore, it is highly unlikely for a solution to settle on an unstable fixed point, because only a cell with initial values of W and C exactly on that fixed point would remain there over time. A cell with values of W and C even slightly different from those values (or upon random fluctuations, which have not been integrated into the model) would settle instead on one of the stable fixed points. In summary, there are three fixed points including one representing CC fate, one representing QC fate and one that is unstable and cannot represent a cell fate; CSC fate is therefore not represented by our single-cell model.

### A C/W multi-cell model achieves stem cell homeostasis

A regulatory network like the one involving W and C, where there are two network components acting antagonistically to each other, is called a switch. A switch forces a decision between two states, because it causes an increase in concentration of one component to result in a decrease in the other, with the eventual outcome that one of them wins (Alon, 2007). In a single cell, this network forces a decision between two cell fates, and it cannot maintain stem cell homeostasis (which requires three cell identities). This behaviour is evident in the solutions of the single-cell model, where there is a fine line between the values of W and C that lead to differentiation and those that lead to a QC fate with no stable region for CSCs between (green box in Fig. 4). This behaviour is fundamentally different from the more robust behaviour of models of the shoot network, because a negative feedback loop controls WUS expression through CLV3, while a mutual repression acts between WOX5 and CLE40 (Brand *et al.*, 2000; Schoof *et al.*, 2000; Stahl *et al.*, 2009; Yadav *et al.*, 2011).

Despite the inability of this model to capture all three cell fates, we wanted to keep the model as small as possible and refrain from adding regulatory components until we had exhausted other options. We were also interested to see if a network with only W and C was capable of describing cell fate. We therefore considered the possibility that this network could still describe stem cell homeostasis if it were implemented in several cells. Each cell could make a cell fate decision as in the single-cell model, but the spatial component could result in a CSC habitable zone where the constant flow of W and C from other cells would result in a tie for W and C regardless of the switch-like nature of the regulatory network. Sophisticated models of the shoot have used 2- and 3-D templates for shoot architecture (Heisler and Jönsson, 2007), as observation of the WUS maximum at the centre of the meristem requires at least two dimensions. In the root, the WOX5, CLE40 and cell fate gradients run in one dimension along the proximal-distal axis. This may make it possible to describe the 3-D effects of WOX5 and CLE40 on cell fate using a one-dimensional model.

**Fig. 5.**
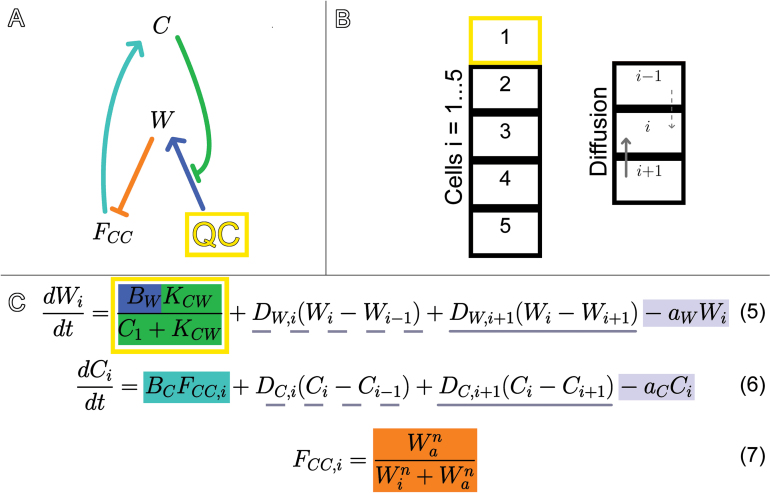
Graphical and mathematical representations of the C/W multi-cell model of cell fate. (A) C represses W (green), which represses F_CC_ (orange). F_CC_ promotes C (teal). W production is confined to the QC cell (yellow). (B) The model simulates a cell column. The cell with index i=1 is the QC (yellow cell), while the model determines the fates of those distal to it. W and C can both diffuse through the cell column and the value of each in cell i are denoted W_i_ and C_i_. (C) Equations 5, 6, and 7. The W production term in the yellow box only applies in the QC cell and W production is restricted by the amount of C signalling to the QC (green). Diffusion terms track the fluxes of W and C between cell i and its proximal neighbour (dotted underline) and between cell i and its distal neighbour (solid underline). A diffusion term is omitted if an index is outside of 1 through 5; there is no flow between the QC and the cell proximal to it and no flow between the 5^th^ cell and the cell distal to it. The value of F_CC_ in cell i (F_CC,i_, orange) determines the fate of cell i and the production rate of C (teal) in cell i. W and C are degraded at constant rates (grey).

To determine if the single-cell model implemented in several cells could describe experimental results, a modified version of the model was implemented in a virtual cell column, with the most proximal cell defined as the W-producing QC cell and the rest allowed to take on either CSC or CC fate. The fate of each cell would still depend on W via F_CC_ as in the single-cell model. With this C/W multi-cell model, W and C could diffuse through the cells, emulating inter-cellular communication. The value of C at the QC determines the production rate of W. The network diagram for the C/W multi-cell model is shown in Fig. 5. We also implemented an alternative model (Supplementary File S1), where values of C at each cell decreased the mobility of WOX5 through that cell, and the results of the alternative model matched the results of the C/W multi-cell model distal to the QC. The alternative C/W multi-cell model, where C regulates W mobility rather than production, is described by the following equations, where the B_W_ term in equation 11 (shown in Fig. 8) only applies in the QC (cell i=1). The equation for F_CC,i_ is identical to equation 7 shown in Fig. 5.

$$\begin{array}{l} \frac{dW_{i}}{dt}=B_{w}+\frac{K_{CW}}{C_{i-1}+K_{CW}}D_{W,i}\left(W_{i}-W_{i-1}\right)\\ \;+\frac{K_{CW}}{C_{i}+K_{CW}}D_{W,i+1}\left(W_{i}-W_{i+1}\right)-a_{w}W_{i}\\ \frac{dC_{i}}{dt}=B_{C}F_{CC,i}+D_{C,i}\left(C_{i}-C_{i-1}\right)\\ \;+D_{C,i+1}\left(C_{i}-C_{i+1}\right)-a_{C}C_{i} \end{array}$$

Parameter values were adjusted by hand, and solutions were obtained for time values until equilibrium was reached. The values of W and C at equilibrium were used to evaluate cell fate in terms of F_CC_. Parameter values were changed to simulate wild-type roots as well as *wox5*, *cle40*, and *wox5/cle40* full loss-of-function mutants, constitutively expressed WOX5, and externally applied CLE40p to wild-type roots. For example, to simulate *wox5* mutants, the production rate of W was set to 0. To simulate constitutive WOX5 expression, the production rates of W in every cell were set to the user-defined maximum W production rate. Addition of CLE40p was simulated by keeping the value of C at a user-defined constant at the QC, where C has its effect on W production. Since CLE40p is applied ectopically and usually in relatively large doses so that the root is flooded, a constant supply of C is more realistic than an increase in production or diffusion rate for C. We assume that the user specifies a high enough number that the level of C at the QC is significantly higher than wild type.

Several initial values of W and C in each cell were tested with the multi-cell models, and this did not change the equilibrium values for any of the realistic parameter sets (all production rates should be greater than or equal to zero, all other parameter values should be greater than 0; m and n should be 1 or greater; and W_a_ should be less than W_b_).

**Fig. 6.**
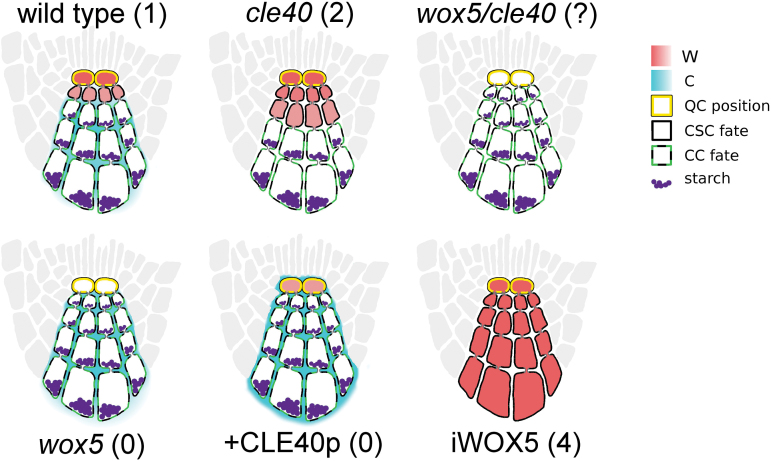
Representation of C/W multi-cell model predictions of CSC fate and W and C localization. The number of expected CSC rows, based on experimental results is shown in parentheses. The model is capable of emulating the results of root phenotypes of wild-type roots, *wox5* mutant roots *cle40* mutant roots, roots with additional synthetic CLE40 (CLE40p) added, and roots expressing *WOX5* in all cells through constitutively expressed *WOX5* with inducible WOX5 function (iWOX5). Results of a *wox5/cle40* double mutant phenotype were also predicted so that the model could be tested later for predictive ability.

C/W multi-cell model predictions are shown in Fig. 6. It was possible to find a range of parameter values that emulated the most common phenotypes of wild type, *wox5* and *cle40* mutants, WOX5 constitutive expression, and addition of CLE40p to wild type.

### The C/W multi-cell model fails to explain *wox5-1/cle40-2* double mutants

The predictive capabilities of a model can be assessed by a validation experiment, a test to see if the model will predict the outcome of an experiment that was not considered in the derivation of the model. To assess the reliability of the C/W multi-cell model, we performed an experiment on *wox5-1/cle40-2* double mutants and compared the results to the model prediction for *wox5/cle40* double mutants.

**Fig. 7.**
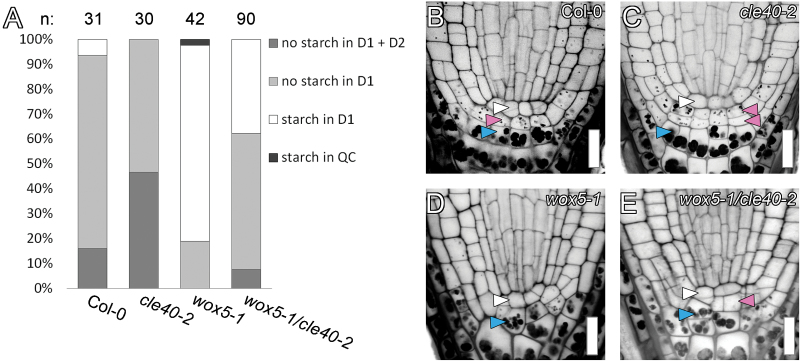
CSC maintenance in Col-0 and *wox5-1*, *cle40-2* and *wox5-1/cle40-2* mutants. (A) Wild-type (Col-0) roots typically maintain stem cells in the D1 layer. Roots lacking CLE40 accumulate more stem cells than wild type, as *cle40-2* mutants more frequently lack starch in D2 as well as D1. Roots lacking WOX5 accumulate less stem cells than the wild type, with a higher frequency of starch in D1. Roots lacking both WOX5 and CLE40 partially rescue the *wox5-1* mutant phenotype. Typical phenotypes are shown for (B) Col-0, and mutants (C) *cle40-2*, (D) *wox5-1* and (E) *wox5-1*/*cle40-2*. White arrows indicate the QC position, pink indicates starch-free cell layers, and blue indicates the first layer with starch. Bars, 30 µm.

Surprisingly, a partial rescue of the *wox5-1* phenotype was observed in the *wox5-1/cle40-2* double mutants (Fig. 7). Since the C/W multi-cell model assumes that W, a WOX5-dependent signal, is necessary for CSC maintenance, it is impossible for the model to correctly predict this result; if there is no WOX5, there is no W, and according to the model there are no CSCs present. The failure of the model indicates that a basic assumption was wrong: WOX5 is not absolutely necessary for CSC maintenance.

Since loss of CLE40 restores CSC fate in *wox5-1* mutants, CLE40 must have an effect on CSC fate that is independent of WOX5. Since biologically active CLE40 is localized to the intercellular space, it cannot directly regulate the processes in the nuclei of target cells to affect their fate; there must be at least one additional component in the CSC regulatory network that affects stem cell fate and is affected by CLE40. Since CLE40 represses CSC fate through this unknown component X, there are two possibilities: either CLE40 promotes expression of X while X represses CSC fate, or CLE40 represses expression of X while X promotes CSC fate from the QC. Neither hypothesis contradicts any experimental results, but the second is simpler. For the first possibility, an ablated QC would result in less WOX5, more differentiation, more CLE40, more X, and further repression of CSC fate by X. If the second possibility were the case, an ablated QC would result both in less WOX5 and less X. Since the latter is the simpler explanation, we tested it further.

### A C/W/X multi-cell model is plausible

**Fig. 8.**
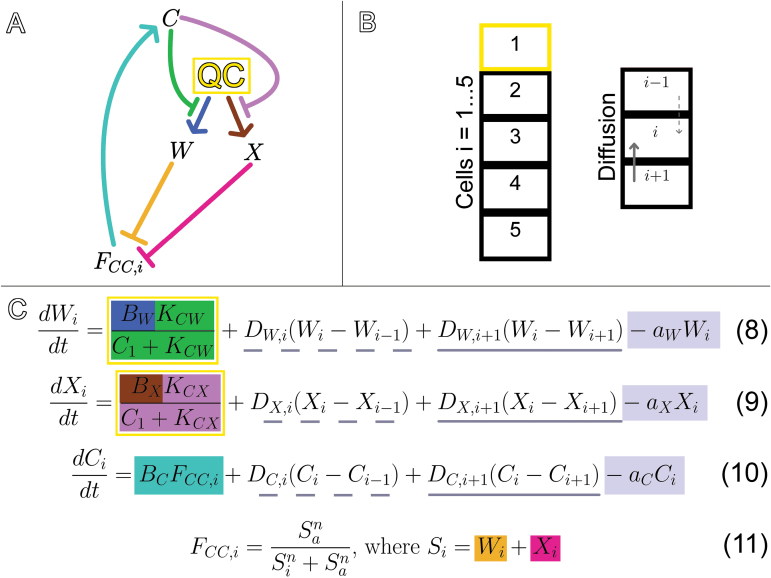
Graphical and mathematical representations of the C/W/X multi-cell model. The C/W/X model was constructed by adding X to the C/W multi-cell model and giving it the same role as W. (A) C represses W (green) and X (purple). W represses F_CC_ (orange), and X represses F_CC_ (pink). F_CC_ promotes C (teal). Production of W and X is confined to the QC cell (yellow). (B) The model simulates a cell column. The cell with index i=1 is the QC (yellow cell), while the model determines the fates of those distal to it. W, X and C can all diffuse through the cell column and the value of each in cell i are denoted W_i_, X_i_ and C_i_. (C) Equations 8, 9, 10, and 11. The production terms of W and X in the yellow box only apply in the QC cell and production of W and X is restricted by the amount of C signalling to the QC (green). Diffusion terms track the fluxes of W, X and C between cell i and its proximal neighbour (dotted underline) and between cell i and its distal neighbour (solid underline). A diffusion term is omitted if the value of i is outside of 1 through 5; there is no flow between the QC and the cell proximal to it and no flow between the 5^th^ cell and the cell distal to it. The value of F_CC_ in cell i (F_CC,i_) is determined by the sum of W_i_ and X_i_. F_CC,i_ determines the fate of cell i and the production rate of C (teal) in cell i. W and C are degraded at constant rates (grey).

**Fig. 9.**
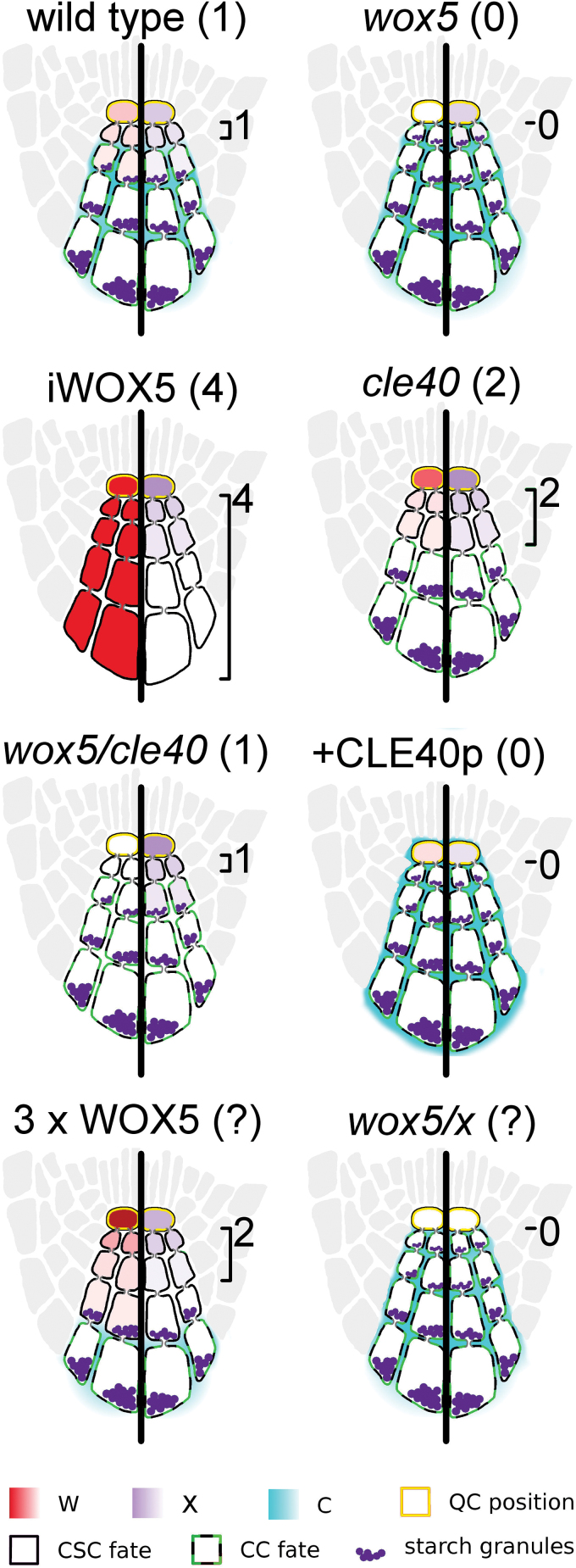
Representation of C/W/X multi-cell model predictions of CSC fate and C/W/X localization. Gradients of W as predicted by the model are shown on the left sides of roots, while X gradients are shown on the right. Expected number of CSC rows, based on experimental results, is shown in parentheses, while those based on model predictions are shown next to the root diagram. The model outcomes can emulate the expected number of rows for wild type, *wox5* mutants, *cle40* mutants, *wox5/cle40* double mutants, constitutively expressed *WOX5* (iWOX5), and the addition of sufficient amounts of CLE40p (+CLE40p). We have also used the model to predict results of experiments that have not yet been completed to aid in later validations of this model. Tripling the W production rate (3× WOX5) is expected to yield more layers of stem cells. In the case of these parameter values, it is expected to result in two layers.

We designed another model, the C/W/X multi-cell model, where X was included with the same role and relationships as W in the C/W multi-cell model (Fig. 8). This C/W/X multi-cell model is able to describe the most common outcomes of all of the experimental results to which we have access, including that of *wox5-1/cle40-2* mutants (Fig. 9). We then used the C/W/X model to predict the phenotype of *wox5/x* mutants. Since the model assumes that either W or X is necessary to maintain CSC fate, those roots are expected to have no CSCs. The model predicts that the number of CSC layers would depend on the rate of *WOX5* expression, so that increasing the rate of W production while still keeping W expression restricted to the QC would result in more stem cell layers due to a higher flux of W to distal cells. In the C/W/X multi-cell model, X functions redundantly with WOX5, perhaps protecting the pluripotent nature of the QC and keeping it from differentiating when WOX5 levels are low. The phenotypes of *wox5-1* mutants suggest that X should be less abundant or less effective at CSC maintenance than the WOX5-dependent signal (W) in the presence of CLE40, since *wox5-1* mutants have less CSCs than *wox5-1/cle40-2* mutants. We simulated this in the model by making the production rate of X less than that of W. Despite the smaller direct effect of X on CSC fate, it had a significant impact on sensitivity of the model to changes in parameters controlling W (Table 2). Due to the buffering activity of X, the model was less sensitive to the production and diffusion rates of W. We conclude that the activity of X allows the network controlling CSC fate to be more robust to perturbations in the levels of WOX5. Starch granules can be found in the QC infrequently in *wox5-1* mutants, but frequently when a substantial amount of synthetic CLE40p is added to *wox5-1* mutants. The model would suggest that the CLE40p is negatively affecting X in this case.

**Table 2.** Parameter values used for simulations Dashes indicate where a parameter was absent from a particular model. Parameters can be perturbed by the amount specified (with all of the others held at the given value), without changing the number and stability of fixed points for the single-cell model or the number of rows of CSCs in each experimental condition for the multi-cell models.

**Parameter**	**1-cell**	**C/W**	**C/W/X**
B_w_	13±31%	34±35%	34±47%
B_c_	3±67%	30±67%	45±67%
B_x_	-	-	25±60%
k_cw_	2±50%	2±50%	2±50%
k_cx_	-	-	2±50%
a_w_	1±50%	2±15%	2±13%
a_c_	1±50%	2±25%	2±25%
a_x_	-	-	2±13%
n	10±90%	2±50%	2±50%
m	10±90%	-	-
W_a_	2±75%	1±25%	-
W_b_	6±58%	-	-
S_a_	-	-	1±20%
D_w_	-	1.2±8%	1±50%
D_c_	-	1±50%	1±25%
D_x_	-	-	1±50%

We now conclude that the roles of WOX5 and CLE40, given their mutually antagonistic nature, are to function as triggers for switches in cell fate. Their interaction does not maintain the robustness of CSC patterning, but discourages cells from staying in a state between CSC and CC fate. As evident from the results of the single-cell model, CSC fate may not be possible without the inter-cellular signalling conducted by WOX5 and CLE40. This result parallels that of the model of the shoot meristem by Yadav *et al.* (2011), which showed the importance of WUS mobility and its resulting gradient to the regulation of stem cell number. Furthermore, it was previously suggested that WOX5 was necessary for CSC fate, so that in *wox5* mutants, CSCs could exist only transiently just after a QC cell division (Sarkar *et al.*, 2007). Using the results of an experiment on *wox5-1/cle40-2* mutants and the inability of the C/W multi-cell model to emulate those results, we have determined that stem cells can be maintained without WOX5 in the absence of CLE40 signalling and, equivalently, that WOX5 is not absolutely necessary for CSC homeostasis. Using the C/W/X multi-cell model, we determined that the existence of another stem-cell promoting factor within the WOX5/CLE40 regulatory network of CSC maintenance is plausible. In the C/W/X model, stem-cell promoting X functions redundantly with WOX5. It would be interesting to see if CLE40 affects SCR expression. Like X, *SCR* expression is independent of WOX5. SCR is known to function redundantly with WOX5 in stem cell maintenance, though to date this has only been demonstrated in the initials proximal to the QC; lack of CSCs is a phenotype of *scr-1* mutants, but this can be explained by lack of WOX5 (Sarkar *et al.*, 2007). Whether X can be found among already-known components of the root stem cell regulatory network (Bennett *et al.*, 2014) like SCR, or among known transcriptional targets of CLE40 (Pallakies and Simon, 2014) remains to be experimentally determined.

## Supplementary data

Supplementary data is available at JXB online.

Supplementary File S1. Text file containing a Matlab program and instructions for its use. The program displays a graphical user interface (GUI), where parameter values can be adjusted and representations of the model output can be acquired.

Supplementary Files S2–S6. Image files required to run the Matlab program.

Supplementary Data
