# Supplementary material for: Mathematical modelling of WOX5- and CLE40-mediated columella stem cell homeostasis in Arabidopsis
Source: J Exp Bot. 2015 May 26;66(17):5375–84. doi: 10.1093/jxb/erv257 (PMC4526915; doi:10.1093/jxb/erv257)
Supplement: Supplementary Data [file supp_66_17_5375__index.html]

Mathematical modelling of WOX5- and CLE40-mediated columella stem cell homeostasis in Arabidopsis — Supplementary Data 

# Mathematical modelling of *WOX5*- and *CLE40*-mediated columella stem cell homeostasis in *Arabidopsis*

## Supplementary Data

Data files

- Supplementary Data - Supplementary Data
- Supplementary Data - Supplementary Data
- Supplementary Data - Supplementary Data
- Supplementary Data - Supplementary Data
- Supplementary Data - Supplementary Data
- Supplementary Data - Supplementary Data
