# Supplementary figures and images for: Mathematical modelling of WOX5- and CLE40-mediated columella stem cell homeostasis in Arabidopsis
Source: J Exp Bot. 2015 May 26;66(17):5375–84. doi: 10.1093/jxb/erv257 (PMC4526915; doi:10.1093/jxb/erv257)

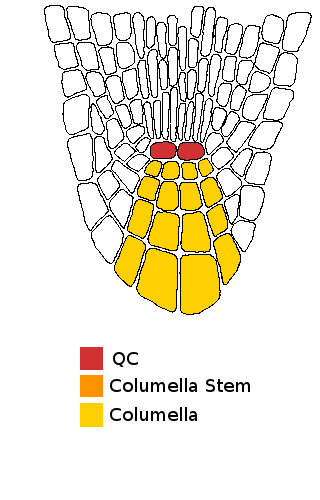

Supplement: Supplementary Data [file supp_erv257_Model3Diagram1.png]

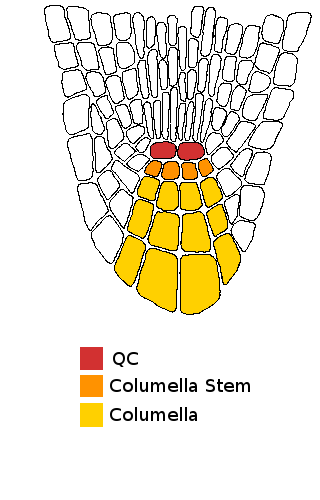

Supplement: Supplementary Data [file supp_erv257_Model3Diagram2.png]

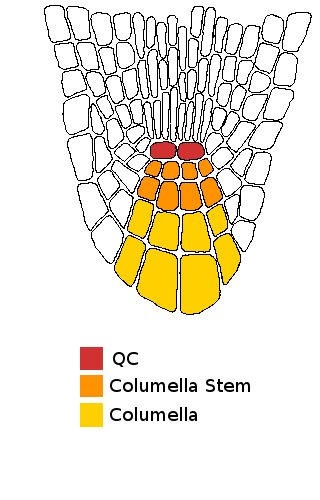

Supplement: Supplementary Data [file supp_erv257_Model3Diagram3.png]

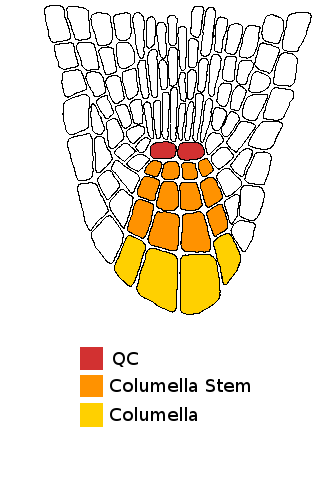

Supplement: Supplementary Data [file supp_erv257_Model3Diagram4.png]

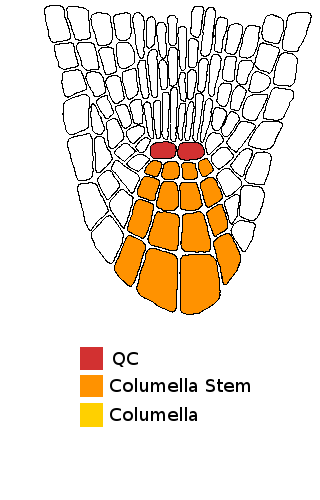

Supplement: Supplementary Data [file supp_erv257_Model3Diagram5.png]
